# Self-focused attention and safety behaviours maintain social anxiety in adolescents: An experimental study

**DOI:** 10.1371/journal.pone.0247703

**Published:** 2021-02-26

**Authors:** Eleanor Leigh, Kenny Chiu, David M. Clark

**Affiliations:** 1 Department of Experimental Psychology, University of Oxford, Oxford, United Kingdom; 2 Department of Psychology, Institute of Psychiatry, Psychology & Neuroscience, King’s College London, London, United Kingdom; University of Sao Paulo Medical School, BRAZIL

## Abstract

**Background:**

Self-focused attention and safety behaviours are both associated with adolescent social anxiety. In adults, experimental studies have indicated that the processes are causally implicated in social anxiety, but this hypothesis has not yet been tested in a youth sample.

**Methods:**

This experiment explored this possibility by asking high and low socially anxious adolescents (N = 57) to undertake conversations under different conditions. During one conversation they were instructed to focus on themselves and use safety behaviours, and in the other they focused externally and did not use safety behaviours. Self-report, conversation partner report and independent assessor ratings were taken.

**Results:**

Self-focus and safety behaviours increased feelings and appearance of anxiety and undermined performance for all participants, but only high socially anxious participants reported habitually using self-focus and safety behaviours.

**Conclusions:**

The findings provide support for the causal role of self-focus and safety behaviours in adolescent social anxiety and point to the potential clinical value of techniques reversing them to treat the disorder.

## Introduction

Social anxiety disorder (SAD) is a common and impairing condition [1, 2], characterised by a low natural recovery rate [3] and adolescent-onset [4]. Developing effective early interventions has the potential to offset the long-term consequences associated with the disorder [5]. Unfortunately, whilst outcomes from standard psychological treatment approaches are promising for most common anxiety problems in youth, they are significantly worse for youth with SAD (e.g. Hudson, Rapee [6], Ginsburg, Kendall [7], Crawley, Beidas [8], Lundkvist-Houndoumadi and Thastum [9]). Establishing psychological mechanisms of social anxiety in youth that can be reversed in treatment may be one way to improve outcomes for this population [10].

Two of the processes that are emphasised in leading cognitive behavioural accounts of social anxiety in adults [11–14] are self-focused attention and safety behaviours. For example, in the model of Clark & Wells (1995), it is proposed that when entering a social or performance situation, socially anxious adults turn their attention inward, to close monitoring of themselves and how they think they are coming across. This is unhelpful because it prevents the individual from discovering how others are responding to them. It also increases awareness of internal information, such as physical feelings of anxiety and mental images, which the individual may then take as confirmation of their negative beliefs. Safety behaviours are strategies, many of which are mental operations, that are intended to prevent or minimise feared outcomes from occurring [15]. For example, an individual may avoid answering a question for fear of getting the answer wrong and appearing stupid. It is suggested that safety behaviours are unhelpful for a number of reasons. They prevent the individual from discovering that the feared outcome was unlikely and/or not catastrophic; they intensify self-focus; they may increase feared symptoms; they can draw attention to feared symptoms; and they can also interfere with the social interaction, for example, not answering a question may be perceived as unfriendly, and so elicit less friendly reactions from others.

Empirical support for the causal role of self-focused attention and safety behaviours in adults comes from experimental studies that have manipulated these variables (see Piccirillo, Taylor Dryman [16] and Norton and Abbott [17] for reviews of safety behaviours and self-focused attention, respectively). Two studies manipulated self-focused attention and safety behaviours simultaneously. In the first study with a sample of adults drawn from an analogue population, McManus, Sacadura, & Clark [18] demonstrated that experimentally manipulating self-focus and safety behaviour use during a conversation task modulated participants’ experience of anxiety and appraisals of their performance, as well as affecting how the participant objectively came across to others. The second study evaluated the effects of this experimental manipulation with a clinical sample of adults as part of cognitive therapy treatment [19]. Patients felt more anxious, thought they appeared more anxious, and came across worse when they focused on themselves and used safety behaviours. This consistent finding was used in subsequent therapy sessions to encourage patients to shift to a more external focus of attention in everyday social interactions and to drop their safety behaviours in order to more effectively test their anxiety provoking expectations.

The question of whether these same processes are present and causally related to social anxiety in adolescents remains relatively unexplored. However, it cannot be assumed that the maintenance processes in adult and adolescent social anxiety are the same, due to reasons such as ongoing cognitive maturation [20], the changing social environment, and heightened sensitivity to social reward [21] during adolescence [10]. A small number of correlational studies examining the association between the two candidate processes and social anxiety symptoms were identified in a recent systematic review of the literature pertaining to the applicability of the Clark & Wells’ cognitive model in adolescents [10]. A moderate association between self-focused attention and social anxiety was reported in three of the four relevant studies [22–25], and a moderate to large association between safety behaviours and social anxiety was found in four studies [22, 23, 26, 27]. Taken together these findings suggest that self-focused attention and safety behaviours co-vary with adolescent social anxiety. Whilst the data is consistent with a causal account, experimental studies are needed in order to comprehensively test the hypothesis. To our knowledge, as yet no studies have been undertaken with adolescents.

Here, we describe an experimental study manipulating self-focus and safety behaviours during a conversation task in high and low socially anxious youth. The effects on self-reported anxiety and performance, conversation-partner ratings, and independent assessor ratings are examined. We tested three main hypotheses in the study: first, that that self-focus and safety behaviours would increase feelings and appearance of anxiety; second, that the detrimental effects of safety behaviours and self-focus would be evident in both high and low socially anxious adolescents, because the safety behaviours employed are typical of those with SAD [26]; and third, that self-focus and safety behaviours would undermine performance, as reflected in more critical judgements by conversation partners and independent observers.

## Methods

### Ethical approvals and consent/assent procedures

The study received ethical approval from the University of Oxford Medical Sciences Division Ethics Committee (CUREC Reference: R54283/RE001). Parental consent and young person assent were obtained for participation in the study.

### Recruitment procedure

Participants were recruited via a screening program undertaken in two secondary schools. All pupils in school years 7–9 (11–14 y) were invited to take part. As part of the screening they completed a measure of social anxiety (the self-report version of the Liebowitz Social Anxiety Scale for Children and Adolescents, LSAS-CA [28]). Those pupils whose scores on the LSAS-CA fell in the top or bottom quartile of the distribution of scores for their year group were invited to take part. The LSAS-CA was repeated at the experimental testing session, and any participant whose score had changed such that they were no longer scoring within their allocated quartile for their year group were not included (*n* = 0).

### Design

High and low socially anxious (SA) participants had two conversations with a naïve conversation partner (gender-matched with participants). In one condition they were instructed to use an agreed set of common safety behaviours and focus on themselves and in the other to focus externally and not use these safety behaviours. The order of condition was counterbalanced within groups and the order of conversation topic was counterbalanced within condition within group. Participants rated their anxiety and how well they thought they performed, and completed manipulation checks after each conversation. Conversation partners rated the participants’ performance, the conversation, and their own anxiety. The conversations were also videotaped and independent assessors rated the interaction.

### Participants

#### Experimental participants

Table 1 provides summary statistics for the high (N = 28) and low (N = 29) SA groups and the total sample. The average age of the sample was 12.75 years (SD = 0.75; min = 11.74 max = 14.58), with no differences between groups. The number of females and males was comparable. As would be expected, the high SA group endorsed significantly higher social anxiety (LSAS-CA) and depression (SMFQ) scores. Their average use of safety behaviours (ASBQ) was significantly higher and they reported being more self-focused (SPWSS item) in social situations.

**Table 1:** Descriptive statistics of participants.

	High Anxiety N = 28	Low Anxiety N = 29	Total N = 57	Statistical Test
	M [SD]	M [SD]	M [SD]	*t* test
Age (y)	12.88 [0.76]	12.64 [0.73]	12.75 [0.75]	*t*(55) = -1.24, *p* = 0.22
LSAS Anxiety^1^	38.46 [14.36]	4.29 [3.80]	21.38 [20.14]	*t*(54) = -12.17, *p*<0.001
LSAS Avoidance^1^	36.32 [14.93]	5.04 [4.34]	20.68 [19.18]	*t*(54) = -10.64, *p*<0.001
LSAS Total^1^	74.79 [28.44]	9.32 [6.90]	42.05 [38.88]	*t*(54) = -11.84, *p*<0.001
SMFQ Total^1^	10.61 [6.68]	2.11 [1.91]	6.36 [6.49]	*t*(54) = -6.47, *p*<0.001
ASBQ^1^	1.42 [0.42]	0.74 [0.44]	1.05 [0.55]	*t*(47) = -5.55, *p*<0.001
SPWSS Item^1^	4.15 [2.28]	3.00 [1.66]	3.57 [2.06]	*t*(52) = -2.21, *p*<0.05
	N	N	N	χ^2^ Test
Gender (F)	18	18	36	χ^2^(1) = 0.30, *p* = 0.86

LSAS: Liebowitz Social Anxiety Scale for Children & Adolescents–Self-Report Version; SMFQ: Short Mood & Feelings Questionnaire; ASBQ: Adolescent Social Behaviour Questionnaire; SPWSS: Social Phobia Weekly Summary Scale.

^1^N = 56 for LSAS and MFQ (High = 28, Low = 28); N = 50 for ASBQ (High = 23, Low = 27); N = 54 for SPWSS item (High = 27, Low = 27).

#### Conversation partners

10 psychology students (50% female) served as the conversation partners. It was explained to them that they were helping with a psychology study about adolescence, but they were naïve to the specific study aims and manipulation. Their average age was 20.50 years (SD: 3.17; range: 16–24), and the average LSAS score was 31.80 (SD: 15.37; range: 3–48).

### Manipulation of self-focused attention and safety behaviours

The instructions for the self-focus and safety behaviours manipulation were consistent with those used in the study of McManus, Sacadura, & Clark (2008). Minor adaptations were made to ensure they were appropriate for adolescents and these were piloted with N = 5. For the ‘With’ self-focus and safety behaviours condition they were as follows: “*Focus on yourself and watch yourself during this conversation*. *Think about how you are coming across to the other person*. *And because you are really thinking about how you are coming across*, *we also want you to be careful about what you say*, *thinking about whether it is the right thing to say*. *And check on that as you go along*, *deciding whether it is good enough to say before you say it*.”. The instructions for the ‘Without’ self-focus and safety behaviours condition were: “*Focus on the other person*. *Don’t think about yourself or how you’re coming across*. *Just be your natural self and respond to whatever the other person says without thinking about that*. *Say whatever comes to mind*. *Absorb yourself in the conversation*, *focusing on the other person*. *As if it is the most interesting conversation you have ever had*.”

### Materials

#### Self-report questionnaires

*Social anxiety symptoms* were measured with the self-report version of the 24 item Liebowitz Social Anxiety Scale for Children and Adolescents, LSAS-CA [28]. Psychometric properties of the scale are sound [29] and internal consistency in the current study was high (Cronbach’s α = .95).

Depression symptoms were measured using the *Short Mood and Feelings Questionnaire* (*SMFQ*) [30]. Internal consistency in the present study was high (Cronbach’s α = 0.93).

The *Child & Adolescent Social Behaviour Questionnaire (ASBQ)* is a 29-item scale of common social safety behaviours (adapted from the adult version of the scale [31]). Respondents are asked how often they tend to use each safety behaviour in social situations when they feel anxious on a 4-point scale (range: 0–3) and a mean score is calculated. This was included to examine whether there were group differences in habitual safety behaviour use. Internal consistency was good in the present study (Cronbach’s α = 0.74).

One item was taken from the *Social Phobia Weekly Summary Scale (SPWSS*; [32]) assessing self-focused attention in difficult social situations (range: 0–8). This was included to examine whether there were group differences in habitual use of self-focused attention in social situations.

*Manipulation Checks* were made with two single item visual analogue scales (VAS). To assess the degree of self-focus, participants were asked to rate “How focused were you on yourself (and how you were coming across) or on the conversation and the other person/people?” after each conversation, from -3 (‘totally self-focused’) to +3 (‘totally focused on the outside’). Participants were also asked to rate the extent to which they had been able to follow the instructions, from 0 (not at all) to 100 (totally).

*Anxiety and Performance Ratings* were made after each conversation on VAS. Participants were asked to rate how anxious they felt and how anxious they thought they appeared (from 0 ‘not at all anxious’ to 100 ‘very severely anxious’). Before the first conversation, participants were asked what their worst fear was for the conversation. After each conversation, they were asked to rate how much they believed their negative prediction to have occurred (from 0 ‘not at all’ to 100 ‘totally’). They were asked how well the conversation went overall (from 0 ‘not at all well’ to 100 ‘really well’). Lastly, they were also asked whether or not they had experienced a negative image of how they came across (yes/no).

*Conversation Partner Ratings* were obtained after each conversation on the following scales: how anxious the participant appeared (from 0 ‘not at all anxious’ to 100 ‘very anxious’); how enjoyable the conversation was (from 0 ‘not at all enjoyable’ to 100 ‘extremely enjoyable’); and how likeable they found the participant (-50 ‘less likeable than average’ to +50 ‘more likeable than average’).

Independent observer ratings of performance were made on a modified version of the Conversation Questionnaire [33]. The questionnaire assesses various aspects of the conversation, such as flow, pauses, and reciprocity, and higher scores indicate a more critical evaluation of the conversation. Minor adaptations were made for use in the present study, to account for an independent rater rather than a conversation partner rater (e.g. “*I interrupted the other person*” was changed to “*The participant interrupted the other person*.”). Items are rated from 0 (‘not at all’) to 8 (‘extremely’). One psychology graduate blind to the condition rated all recorded interactions. The rater demonstrated excellent interrater reliability on a subsample of 50% of the videos rated by the lead author (*ICC* = .95, 95% CI: .93-.96).

### Procedure

Testing took place in school hours. Before beginning, it was explained to conversation partners that they would be taking part in brief conversations with young people. They were asked to imagine that they were meeting them for the first time at a family party or social event. They were instructed not to talk about the experiment during the conversation. It was explained to participants that they were participating in a study to find out how young people think and feel in social situations. Participants initially completed the self-report questionnaires. Participants were allocated to the ‘With’ self-focused attention and safety behaviours or ‘Without’ in counterbalanced order within groups. Prior to the first conversation, participants were asked to identify their worst fears for a conversation with a stranger and what safety behaviours they would normally use to avoid these feared outcomes. They then underwent the self-focused attention and safety behaviour manipulation. Participants were asked to continue following the instructions throughout the following conversation with the conversation partner whom they had not met before. Participants were given a conversation topic (either ‘Hobbies’ or ‘Holidays’, these were assigned in counterbalanced order within conditions within groups). The conversation partner, who was unaware of the study design, was also given the conversation topic. The participant and conversation partner were introduced to one another and the experimenter sat discreetly in the room during the conversation. Each conversation lasted 5 minutes. After each conversation, participants completed *Manipulation Check* items, *Anxiety and Performance Ratings* and the conversation partner completed *Conversation Partner Ratings*. At the end of the session participants were thanked for their time and debriefed.

## Results

### Data analysis

A series of linear mixed models was conducted on the self-report, conversation-partner report, and independent assessor ratings. Condition and group were included as fixed factors, and the interaction between condition and group. Conversation partner was included as a random effect. To test for possible condition order effects, all models were first run with this variable included as a fixed factor, with the two- and three-way interactions. The covariance structure matrix was unstructured, and Maximum Likelihood was used for model estimation. All analyses were conducted using R [34]. Significance was considered at *p* < .05, two-tailed.

Means and standard deviations for the variables included in the following analysis are presented in Table 2.

**Table 2:** Means and standard deviations for the high and low social anxiety groups in the ‘With’ and ‘Without’ conditions.

Measure	Social Anxiety Group	Condition	
		With safety behaviours and self-focus (N = 57)	Without safety behaviours and self-focus (N = 57)
*Manipulation Checks*			
Self-focus (-3 –+3)			
	Low	-0.47 (0.88)	1.78 (1.18)
	High	-1.45 (1.23)	0.66 (1.27)
Followed instructions (0–100)			
	Low	58.21 (21.16)	82.86 (15.88)
	High	65.36 (21.17)	60.00 (18.81)
*Participant Anxiety and Performance Ratings*			
Feel anxious (0–100)			
	Low	35.69 (21.46)	10.48 (12.00)
	High	66.61 (16.50)	41.79 (23.26)
Look anxious (0–100)			
	Low	43.45 (24.06)	11.55 (15.55)
	High	64.11 (18.76)	39.82 (21.58)
Belief (0–100)			
	Low	23.55 (25.57)	9.14 (16.26)
	High	57.14 (28.46)	32.68 (24.66)
How well the conversation went (0–100)			
	Low	61.83 (23.94)	83.59 (12.34)
	High	43.21 (22.29)	55.89 (18.91)
*Perceiver Ratings*			
Participant looked anxious (0–100)			
	Low	45.17 (26.41)	28.97 (23.35)
	High	67.50 (28.63)	50.36 (27.01)
Enjoyed conversation (0–100)			
	Low	67.24 (21.20)	76.90 (15.83)
	High	49.64 (25.46)	64.29 (23.32)
Liked the participant (-50 –+50)			
	Low	19.14 (16.48)	26.21 (12.65)
	High	11.07 (16.85)	17.50 (16.24)
*Observer Ratings*			
Conversation Questionnaire (0–96)			
	Low	20.16 (13.64)	9.89 (10.62)
	High	40.24 (16.69)	24.33 (13.93)

### Linear mixed-effects models analyses

Results of linear mixed models excluding condition order as a fixed factor are presented because no significant order effects or interactions were observed.

#### Manipulation checks

As shown in Table 3, participants were more self-focused in the ‘With’ condition, as intended. High, compared to low, socially anxious participants were more self-focused in general. Participants were more able to follow instructions in the ‘Without’ condition, and there was a significant interaction effect between condition and group. Post-hoc Tukey HSD tests showed that low socially anxious adolescents reported following the instructions more in the ‘Without’ condition than in the ‘With’ condition (*p* < .001). There were no significant differences in compliance for high socially anxious adolescents between conditions (*p* = .69). The high socially anxious adolescents reported significantly lower compliance in the ‘Without’ condition (*p* < .001) compared to the low socially anxious participants.

**Table 3:** Effects of experimental condition and social anxiety group on manipulation checks.

	Self-focus			Followed instructions		
Predictors	*b*	CI	*p*-value	*b*	CI	*p*-value
Intercept	-0.36	-0.62 – -0.10	0.007	-0.41	-0.79 – -0.02	0.037
Condition	1.35	1.00 – 1.70	<0.001	1.15	0.71 – 1.58	<0.001
Group	-0.60	-0.96 – -0.24	0.001	0.27	-0.19 – 0.73	0.248
Condition x Group	-0.08	-0.58 – 0.42	0.753	-1.40	-2.02 – -0.77	<0.001
Random Effects						
σ^2^	0.47			0.72		
τ_00_	0.01 _PerceiverID_			0.11 _PerceiverID_		
ICC	0.02			0.13		
N	11 _PerceiverID_			11 _PerceiverID_		
Observations	114			114		
Marginal R^2^ / Conditional R^2^	0.528 / 0.539			0.212 / 0.315		

CI = 95% confidence interval

Marginal R^2^ = variance explained by random and fixed effects

Conditional R^2^ = variance explained by fixed effects

Perceiver ID = Conversation partner ID

#### Self-report anxiety and performance ratings

Table 4 shows that there was a main effect of condition across all anxiety and appraisal ratings, with participants in the ‘With’ condition as feeling more anxious, believing they appeared more anxious, believing more strongly their feared prediction had occurred, and thinking they did less well. There was also a main effect of group across all the ratings, with high socially anxious adolescents feeling more anxious and believing they appeared more anxious, believing more strongly that their feared prediction had come true, and thinking that they had come across less well. No interaction effects were significant.

**Table 4:** Linear mixed model results for self-report ratings.

	Felt anxious			Looked anxious			Belief			How well the conversation went		
Predictors	*B*	CI	*p*-value	*b*	CI	*p*-value	*b*	CI	*p*-value	*b*	CI	*p*-value
Intercept	-0.08	-0.35 – 0.18	0.527	0.17	-0.14 – 0.48	0.281	-0.25	-0.56 – 0.07	0.126	-0.02	-0.37 – 0.34	0.916
Condition	-0.92	-1.27 – -0.57	<0.001	-1.16	-1.53 – -0.80	<0.001	-0.49	-0.90 – -0.07	0.021	0.89	0.50 – 1.28	<0.001
Group	1.12	0.76 – 1.48	<0.001	0.73	0.35 – 1.11	<0.001	1.14	0.72 – 1.57	<0.001	-0.69	-1.10 – -0.28	0.001
Condition x Group	0.01	-0.48 – 0.51	0.956	0.28	-0.24 – 0.80	0.294	-0.34	-0.93 – 0.25	0.258	-0.37	-0.93 – 0.19	0.192
Random Effects												
σ^2^	0.46			0.50			0.64			0.58		
τ_00_	0.02 _PerceiverID_			0.06 _PerceiverID_			0.03 _PerceiverID_			0.11 _PerceiverID_		
ICC	0.03			0.11			0.04			0.16		
N	11 _PerceiverID_			11 _PerceiverID_			11 _PerceiverID_			11 _PerceiverID_		
Observations	114			114			114			114		
Marginal R^2^ / Conditional R^2^	0.528 / 0.543			0.451 / 0.512			0.346 / 0.373			0.324 / 0.434		

CI = 95% confidence interval

Marginal R^2^ = variance explained by random and fixed effects

Conditional R^2^ = variance explained by fixed effects

Perceiver ID = Conversation partner ID

After each conversation, participants were asked whether they had experienced a negative self-image. In the ‘With’ condition, 17/28 (61%) of the high social anxiety group and 11/29 (38%) of the low social anxiety group experienced a negative image. In the ‘Without’ condition, 4/28 (14%) and 0/29 (0%) of the high and low groups, respectively, reported a negative image. McNemar’s test indicated that significantly more negative images were experienced in the ‘With’ compared to the ‘Without’ condition (*p* < .001).

#### Conversation partner ratings

Table 5 shows that there was a main effect of condition on two of the three of the conversation partner report ratings, with conversation partners rating participants in the ‘Without’ condition as looking less anxious and more likeable than those in the ‘With’ condition. The effect of condition on how enjoyable conversation partners rated the interaction was approaching significance (*p* = .077). There was a main effect of group across all the ratings, with conversation partners rating high socially anxious participants as looking more anxious, less likeable, and the conversation as less enjoyable. No other main effects or interaction effects were significant.

**Table 5:** Linear mixed model results for conversation partner ratings.

	Participant looked anxious				Enjoyed conversation			Liked participant			
Predictors	*b*	CI	*p*-value	*b*		CI	*p*-value		*b*	CI	*p*-value
Intercept	-0.08	-0.44 – 0.27	0.648	0.04		-0.33 – 0.42	0.824		-0.07	-0.51 – 0.36	0.742
Condition	-0.55	-1.00 – -0.10	0.016	0.41		-0.04 – 0.86	0.077		0.43	0.01 – 0.85	0.043
Group	0.82	0.35 – 1.28	0.001	-0.78		-1.25 – -0.30	0.001		-0.56	-1.00 – -0.11	0.014
Condition x Group	-0.03	-0.67 – 0.61	0.922	0.21		-0.44 – 0.86	0.522		-0.04	-0.64 – 0.56	0.898
Random Effects											
σ^2^	0.76				0.78			0.66			
τ_00_	0.05 _PerceiverID_				0.08 _PerceiverID_			0.26 _PerceiverID_			
ICC	0.06				0.09			0.28			
N	11 _PerceiverID_				11 _PerceiverID_			11 _PerceiverID_			
Observations	114				114			114			
Marginal R^2^ / Conditional R^2^	0.230 / 0.280				0.176 / 0.250			0.122 / 0.367			

CI = 95% confidence interval

Marginal R^2^ = variance explained by random and fixed effects

Conditional R^2^ = variance explained by fixed effects

Perceiver ID = Conversation partner ID

#### Observer ratings

Table 6 presents findings from the linear mixed models for observer ratings. Conversations were rated more critically in the ‘With’ condition compared to the ‘Without’ condition. There was also a main effect of group on observer ratings, with high socially anxious adolescents receiving more critical ratings than low socially anxious adolescents. The interaction effect was statistically non-significant.

**Table 6:** Linear mixed model results for observer ratings.

	Conversation questionnaire		
Predictors	*b*	CI	*p*-value
Intercept	-0.25	-0.64 – 0.14	0.211
Condition	-0.58	-1.08 – -0.08	0.023
Group	1.14	0.64 – 1.65	<0.001
Condition x Group	-0.32	-1.01 – 0.36	0.357
Random Effects			
σ^2^	0.60		
τ_00_ _PerceiverID_	0.04		
ICC	0.06		
N _PerceiverID_	8		
Observations	79		
Marginal R^2^ / Conditional R^2^	0.377 / 0.416		

CI = 95% confidence interval

Marginal R^2^ = variance explained by random and fixed effects

Conditional R^2^ = variance explained by fixed effects

Perceiver ID = Conversation partner ID

## Discussion

The findings of this experimental study are consistent with the hypothesis that self-focused attention and safety behaviours are relevant to the maintenance of adolescent social anxiety. Participants felt more anxious and thought they looked more anxious in the ‘With’ compared to the ‘Without’ condition. They also thought they came across worse and that their negative predictions were more likely to have occurred. To a certain extent their fears were realised, in that the conversation partner and the independent observer rated them more critically, in terms of looking more anxious and being less likeable, when self-focused and using safety behaviours.

It is interesting to note that the detrimental effects of self-focus and safety behaviours were observed in both high and low anxiety groups. The finding indicates that focusing on oneself and using safety behaviours in social situations is unhelpful for adolescents in general, whether or not they are socially anxious. However, when we compare ratings of safety behaviour use and self-focus in social situations day-to-day, significant group differences were observed. It therefore seems that whilst using safety behaviours and focusing internally is unhelpful for all adolescents, young people who are more socially anxious tend to use them more in everyday life, and so this will mean that the adverse effects will only manifest for this population. Extending from this observation is the question of why some individuals adopt these psychological processes more than others. Longitudinal studies examining the temporal associations between negative social beliefs and safety behaviours and self-focused attention could test the hypothesis put forward by cognitive accounts of social anxiety that it is negative social schema that motivate the use of safety behaviours and self-focus (e.g. [12]).

Our findings provide evidence for mechanisms of adolescent social anxiety: safety behaviours and self-focus. We cannot draw conclusions about the extent to which effects are driven by self-focused attention and/or the use of safety behaviours, because both processes were manipulated together. This is because in reality it is difficult to conceive of an individual using common safety behaviours such as monitoring how they are coming across and rehearsing what they are going to say *without* focusing on themselves. Whilst it is difficult to manipulate safety behaviours without manipulating self-focus, future studies that examine the effect of manipulating focus of attention only during a social interaction task would be useful. This limitation perhaps highlights the interconnectedness of the processes [35] emphasised in the dominant cognitive behavioural models of social anxiety in adults (e.g. [11, 12]). Support for the idea that the processes are reciprocally related also comes from this study (see also [36]). Participants were asked whether or not they had experienced an image or impression of how they came across during each conversation. Despite no instructions relating to mental imagery, almost half of all participants (49%) experienced a spontaneous negative image of how they looked when they were self-focused and using safety behaviours. One participant described an image of themselves as “*The weird kid sitting in the corner*”, another as “*Sweating*, *blushing*, *uptight*, *spoilt*, *posh*”. This compares to only 7% (4/59) in the ‘Without’ condition. The finding aligns with the suggestion that self-focus increases awareness of internally generated information such as negative images and impressions [37].

Participants were instructed to use a common set of safety behaviours, based on those used in the study of McManus et al [18]. However, according to cognitive accounts of social anxiety, safety behaviours are highly idiosyncratic to individuals and to their specific fears. As a result, we cannot conclude from our findings that the use of any safety behaviours is detrimental, but rather that the use of this particular set of safety behaviours is. However, the specific instructions that we used for the safety behaviour manipulation were based on safety behaviours that are particularly commonly reported by both socially anxious adults [18, 19, 38] and adolescents [10, 27, 39]. These safety behaviours are also strongly correlated with self-reported social anxiety in those samples when the correlations are based on naturalistic use [22, 23]. In addition to this limitation, we note that conversation partners were on average eight years older than participants, which may limit generalisability of our findings to an extent, and future studies with similar-aged peers as conversation partners would be useful. Furthermore, single item measures are prone to random measurement error and so future studies could include multi-item measures.

Although the sample was not drawn from a clinical population, the average score of high social anxiety participants’ on a measure of social anxiety (LSAS-CA) was high (74.79 [28.44]) and the participant with the lowest LSAS-CA scored 29, which is above the suggested threshold (22.5) for distinguishing adolescents with social phobia and healthy controls [29]. Therefore, the findings of this study may well be pertinent to the task of developing therapeutic techniques for adolescents with SAD. Cognitive Therapy for adult SAD [40], includes a *self-focused attention and safety behaviours experiment* similar to the one in this study in session 2 of the treatment. Therapists use it to help patients discover the unintended consequences of the processes. This demonstration is later followed by *attention retraining* to help patients reduce self-focus and become more externally focused in social situations; and *behavioural experiments* to help patients test out their predictions in feared situations whilst dropping their safety behaviours and focusing on other people and their reactions. Our results suggest that also incorporating the *self-focused attention and safety behaviours* experiment and linked procedures into CBT protocols for adolescent social anxiety may be beneficial. Consistent with this suggestion, two outcome studies that used versions of the adult Cognitive Therapy for SAD protocol with adolescents have shown promising results [41, 42].

This study indicates that the use of safety behaviours and self-focus in social situations is unhelpful. The processes are problematic in a number of ways, including increasing anxiety, maintaining negative self-perceptions, prompting negative mental imagery, and contaminating social performance. This is consistent with cognitive models of social anxiety in adults that suggest specific psychological processes (social cognitions, negative imagery, self-focused attention, and safety behaviours) create interlocking reciprocal links that lock individuals into a cycle of social anxiety. Our study suggests that similar processes may operate in adolescents and point to potential opportunities for intervention.

## Abbreviations

SA: Social anxiety
SAD: Social Anxiety Disorder
VAS: Visual Analogue Scales
